# Supplementary material for: The causal relationship between the human gut microbiota and pyogenic arthritis: a Mendelian randomization study
Source: Front Cell Infect Microbiol. 2024 Nov 26;14:1452480. doi: 10.3389/fcimb.2024.1452480 (PMC11629706; doi:10.3389/fcimb.2024.1452480)
Supplement: Supplementary file 1 [file DataSheet1.zip › mendelian test/ebi-a-GCST90027774.csv_forest.pdf]

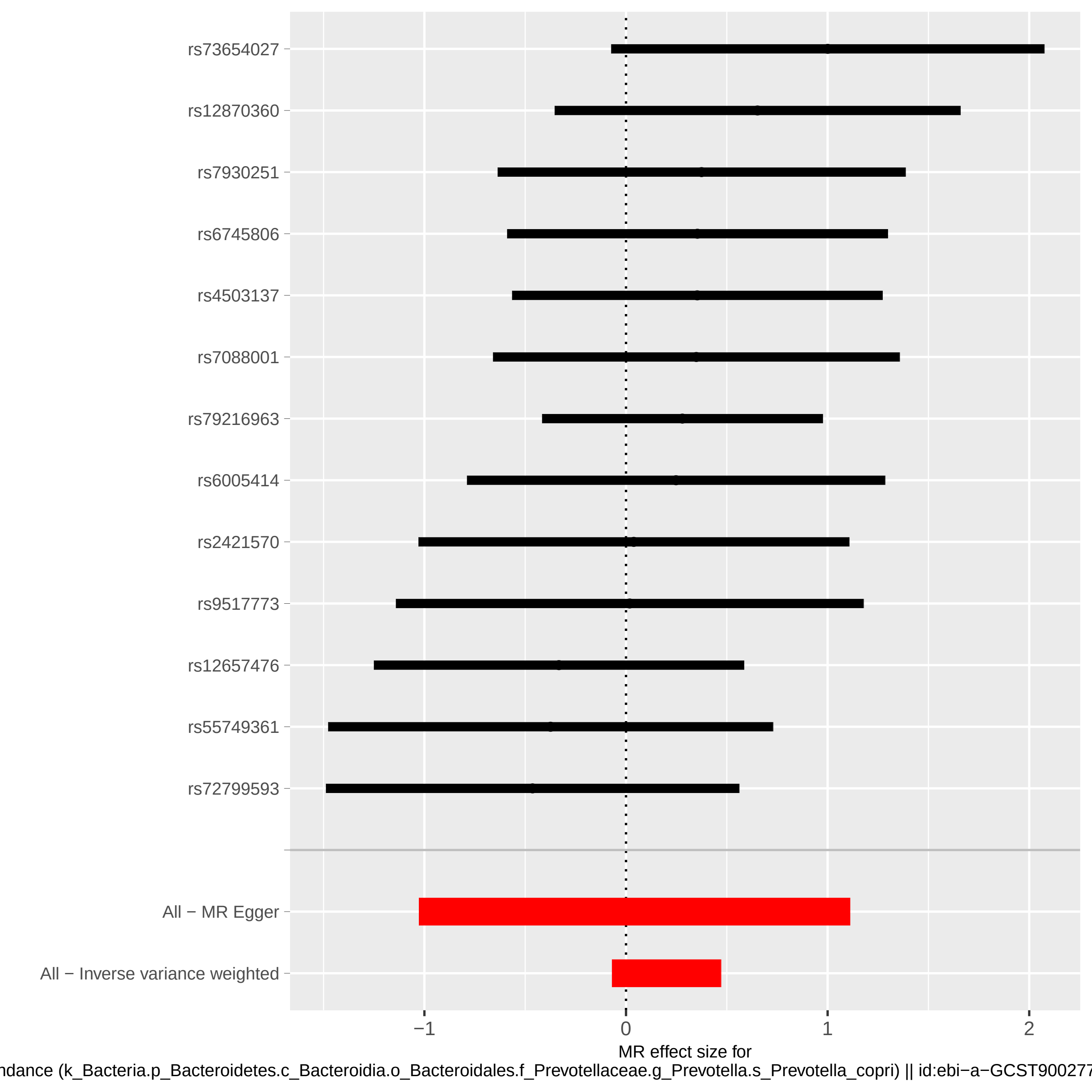
rs73654027
rs12870360
rs7930251
rs6745806
rs4503137
rs7088001
rs79216963
rs6005414
rs2421570
rs9517773
rs12657476
rs55749361
rs72799593
All − MR Egger
All − Inverse variance weighted
−1
0
1
2
MR effect size for
ndance (k_Bacteria.p_Bacteroidetes.c_Bacteroidia.o_Bacteroidales.f_Prevotellaceae.g_Prevotella.s_Prevotella_copri) || id:ebi−a−GCST90027
